# Artificial Heat in Hospitals

**Published:** 1924-11

**Authors:** 


### ARTIFICIAL HEAT IN HOSPITALS

The outstanding advantage of warming by electricity is that the heat can be taken to the object to be warmed, while with every other method the object has to go or be taken to the source of heat. A patient lying in bed, for instance, can have warmth delivered to any part of his body as he lies there, without any exertion on his part. This method possesses the further very useful property, that by means of suitable appliances the heat delivered can be regulated as may be required, within certain fairly wide limits. When an electric current flows through a conductor, to use the popular belief with regard to the current, the energy carried by the current is converted into heat in a certain definite manner. The number of heat units delivered to the conductor in any given time can be calculated, and if the rate at which the heat is dissipated is known, the increase of temperature of the conductor can also be calculated in any given time.

### Methods of Application.

There are two methods of applying heat by means of electric currents at present—by what are practically electric incandescent lamps, and by means of wires drawn from metals that have been designed not to increase their electrical resistance to any appreciable extent with a rise of temperature. With nearly all substances the electrical resistance of the substance increases in a certain definite ratio, as the temperature increases ; but there are a few substances in which the opposite rules. The resistance falls as the temperature rises, and by judiciously alloying, metals have been produced the electralic resistance of which remains practically constant over comparatively wide ranges of temperature ; and a number of applications of these alloys have been made for use with electrical heating. An examination of the formulæ will show that, if that were not so, the resistance of the heating apparatus would increase as the current flowed through it, altering the working of the apparatus. As long as the electrical resistance of the apparatus remains practically constant, the heating effect of a given current strength should remain constant. For practical purposes, it may be taken that in modern apparatus this is so. This applies to heating apparatus in which metal wires are employed formed of one of the alloys referred to. With what are really large electric incandescent lamps it does not apply.

### Warming the Patient's Body.

Electrically heated elements may be carried by many forms of garments. For instance, I understand that airmen are protected from the very low temperatures they have to encounter by means of electrically warmed garments—coats, trousers, gloves, etc.—and probably some of the feats that have been accomplished by airmen would have been impossible but for this. It is quite practicable to apply heat electrically to any part of a patient by enclosing wires in the garments he is wearing, or in portable apparatus that can be slipped into the bed, or can be inserted in the garments, even while the patient is up, resting in a chair, or on a couch, for instance. It would even be quite practicable to provide electrically warmed garments to patients standing or walking if either the electrical apparatus concealed in their garments can be connected to a source of electric current (this is quite practicable), or the patient can carry a source of current about with him. This is also practicable to a limited extent. There are primary and secondary batteries on the market of sufficiently light weight to be carried by a patient for a limited time that will furnish sufficient current to heat part of the patient's body for the time the patient is able to carry them.

### The Weak Point of Electrical Heating.

The weak point of electrical heating is the possibility of the heating elements being burnt out while the patient is using them. This should be avoided easily by care. Whenever an electric current flows through a conductor, the mere passage of the current, added to the changes of temperature to which the conductor is subject, tends to render it brittle, with the result that, generally at some awkward time, the conductor parts—breaks practically—the current ceasing to flow, and the heat stopping immediately. Unfortunately this is not the whole trouble. A spark nearly always passes between the ends of a conductor that is parted, throug which a current has been flowing previously to the parting ; and the spark often leads to other troubles. It may, for instance, lead to the ignition of inflammable substances in the neighbourhood. This can, of course, be easily guarded against, but it is wise to bear the danger in mind.

